# A Comparison of Parenteral Phenobarbital vs. Parenteral Phenytoin as Second-Line Management for Pediatric Convulsive Status Epilepticus in a Resource-Limited Setting

**DOI:** 10.3389/fneur.2019.00506

**Published:** 2019-05-15

**Authors:** Richard J. Burman, Sally Ackermann, Alexander Shapson-Coe, Alvin Ndondo, Heloise Buys, Jo M. Wilmshurst

**Affiliations:** ^1^Division of Paediatric Neurology, Department of Paediatrics and Child Health, Red Cross War Memorial Children's Hospital, University of Cape Town, Cape Town, South Africa; ^2^Faculty of Health Sciences, University of Cape Town Neuroscience Institute, Cape Town, South Africa; ^3^Ambulatory and Emergency Services, Department of Paediatrics and Child Health, Red Cross War Memorial Children's Hospital, University of Cape Town, Cape Town, South Africa

**Keywords:** Africa, convulsive status epilepticus, management, pediatrics, phenobarbital

## Abstract

**Introduction:** Pediatric convulsive status epilepticus (CSE) which is refractory to first-line benzodiazepines is a significant clinical challenge, especially within resource-limited countries. Parenteral phenobarbital is widely used in Africa as second-line agent for pediatric CSE, however evidence to support its use is limited.

**Purpose:** This study aimed to compare the use of parenteral phenobarbital against parenteral phenytoin as a second-line agent in the management of pediatric CSE.

**Methodology:** An open-labeled single-center randomized parallel clinical trial was undertaken which included all children (between ages of 1 month and 15 years) who presented with CSE. Children were allocated to receive either parenteral phenobarbital or parenteral phenytoin if they did not respond to first-line benzodiazepines. An intention-to-treat analysis was performed with the investigators blinded to the treatment arms. The primary outcome measure was the success of terminating CSE. Secondary outcomes included the need for admission to the pediatric intensive care unit (PICU) and breakthrough seizures during the admission. In addition, local epidemiological data was collected on the burden of pediatric CSE.

**Results:** Between 2015 and 2018, 193 episodes of CSE from 111 children were enrolled in the study of which 144 met the study requirements. Forty-two percent had a prior history of epilepsy mostly from structural brain pathology (53%). The most common presentation was generalized CSE (65%) caused by acute injuries or infections of the central nervous system (59%), with 19% of children having febrile status epilepticus. Thirty-five percent of children required second-line management. More patients who received parenteral phenobarbital were at a significantly reduced risk of failing second-line treatment compared to those who received parenteral phenytoin (*RR* = 0.3, *p* = 0.0003). Phenobarbital also terminated refractory CSE faster (*p* < 0.0001). Furthermore, patients who received parenteral phenobarbital were less likely to need admission to the PICU. There was no difference between the two groups in the number of breakthrough seizures that occurred during admission.

**Conclusion:** Overall this study supports anecdotal evidence that phenobarbital is a safe and effective second-line treatment for the management of pediatric CSE. These results advocate for parenteral phenobarbital to remain available to health care providers managing pediatric CSE in resource-limited settings.

**Attachments:** CONSORT 2010 checklist

**Trial registration:** NCT03650270

**Full trial protocol available:** https://clinicaltrials.gov/ct2/show/NCT03650270?recrs=e&type=Intr&cond=Status+Epilepticus&age=0&rank=1

## Key point box

- Parenteral phenobarbital as a second-line treatment terminates refractory pediatric convulsive status epilepticus more effectively than parenteral phenytoin
- Parenteral phenytoin for convulsive status epilepticus is associated with increased admission rates to pediatric intensive care
- Midazolam infusion for convulsive status epilepticus increases the demand on the intensive care units

## Introduction

The management of status epilepticus (SE) continues to be a significant challenge in modern epileptology. This enduring and self-perpetuating seizure activity can have a plethora of semiologies with generalized convulsive SE (CSE) being the most common. Seizures that do not self-terminate within 5 min (or recur repeatedly) are less likely to do so without therapeutic intervention (1). Therefore, the practical definition of SE is any seizure that is >5 min in duration or multiple discrete seizures between which there is no extended period of recovery (2).

Children commonly present with CSE to pediatric emergency medical centers (3–5). While epidemiological data on pediatric CSE are lacking globally, this is particularly true for resource-limited settings such as in sub-Saharan Africa. CSE remains an important medical emergency as without effective management, neurological sequelae and mortality can ensue (5, 6). In addition, SE poses a significant cost to healthcare institutions due to the intensive management and monitoring that these patients require (7). Furthermore, all the current treatment guidelines for CSE are primarily based on evidence gathered from high-income, resource-equipped countries (8–10). In contrast, there remains a lack of robust data guiding the management of SE, especially in children and in those patients based in Africa.

The treatment guidelines that are available for CSE recommend the use of benzodiazepines as the first line-agents (9). These include either diazepam, lorazepam or midazolam as they have comparable antiseizure efficacy (11). However, in a subset of patients, mostly children, these agents are ineffective (12). When this occurs, these patients require second-line agents which are typically more difficult to administer and have a greater potential for severe adverse effect profile (13). There is currently poor evidence regarding which second-line agent to use in pediatrics. Historically parenteral phenytoin is used, but there is little evidence to support its use. However, there are multi-center randomized-controlled studies currently underway in high-income settings aimed at determining the optimal second-line intervention for pediatric CSE. These include: the Emergency Treatment with Levetiracetam or Phenytoin in Status Epilepticus (EcLiPSE) study based in the United Kingdom and recruiting participants from 6 months to 18 years of age (14); the Established Status Epilepticus Treatment Trial (ESETT) based in the United States which is comparing parenteral fosphenytoin, valproate and levetiracetam in patients older than 2 years (15); and the Convulsive Status Epilepticus Pediatric Trial (ConSEPT) study in New Zealand comparing parenteral formulations of phenytoin against levetiracetam in patients between 3 months and 16 years of age (16). While these studies will provide important data, their results cannot be easily extrapolated or translated to patients in Africa who face significant healthcare restraints and do not have ready access to the newer, more expensive agents like parenteral levetiracetam.

Within our area of practice, if parenteral phenobarbital and phenytoin is ineffective in terminating refractory CSE, typically the next line of intervention is a parenteral infusion of midazolam (17–20). However, administering these agents usually requires additional infrastructure (e.g., infusion pumps) and admission to an intensive care unit due to the significant risk of cardiopulmonary depression. Access to pediatric intensive care (PICU) unit is limited across Africa and therefore the use of a midazolam infusion is viewed with caution.

Phenobarbital is widely used across Africa as a low cost and effective addition to the antiseizure arsenal, particularly in pediatric epilepsies (21). Currently, there exist only a few studies demonstrating how the use of parenteral phenobarbital can be effective in terminating CSE in neonates, pediatrics and adults (22–24). Our experience at the Red Cross War Memorial Children's Hospital (RCWMCH) in Cape Town, South Africa is that parenteral phenobarbital is an effective and preferred treatment for refractory pediatric CSE. Moreover, our impression is that repeated doses of parenteral phenobarbital is both more effective and safer than the traditional approach of using parenteral phenytoin followed by a midazolam infusion. However, to date we have not been able to provide evidence to support this anecdotal evidence. Furthermore, validating the use of parenteral phenobarbital is becoming increasingly important by the frequent limited access across sub-Saharan Africa and worldwide which often results in it needing to be imported via complex regulatory channels (25–27).

The aim of this study was to demonstrate the efficacy of parenteral phenobarbital (PHB) as second-line management for pediatric CSE refractory to benzodiazepines. We compared two treatment protocols, one containing PHB as the second-line agent and the other using parenteral phenytoin (PHY). We compared these agents by looking at how effective they were in terminating CSE (primary outcome), whilst also comparing differences in the need for PICU admission and breakthrough seizures (secondary outcomes). In addition, for patients that fail second-line treatment, we reviewed the use of repeated parenteral boluses of PHB vs. the use of a midazolam (MDZ) infusion. Furthermore, our study also collected local epidemiological data on the burden of pediatric CSE.

## Materials and Methods

### Study Design and Inclusion Criteria

This was an open-label single-center randomized parallel clinical trial which was conducted at the RCWMCH and ran between March 2015 and March 2018. The study was stopped at this time as access to PHB became limited. All children from 1 month to 15 years of age who presented with CSE needing therapeutic intervention were entered into this study by the attending medical staff. Study data were collected using REDCap hosted by the University of Cape Town's eResearch Center and the study was approved by the UCT Human Research Ethics Committee (UCT HREC 297/2005). At recruitment, initially verbal consent was obtained from the child's parent or legal guardian for the intervention as it was part of routine medical care at RCWMCH. After the CSE had been terminated, full written consent was obtained from the parents or legal guardian in order to use the child's clinical data for research purposes. In addition, if medical records were incomplete or missing, these children were also excluded. Children who were on chronic treatment with phenobarbital or phenytoin and or had received intravenous phenobarbital or phenytoin prior to admission were excluded from the study.

### Definitions and Data Collection

CSE was defined as any convulsive seizure that lasted longer than 5 min (“continuous”) or multiple discrete seizures between which there was no extended period of recovery between events (“intermittent”) (2). The time of CSE onset was defined as the time provided by the caregiver who accompanied the child. The time to admission and to treatment were recorded by the attending medical emergency unit staff. If children were admitted multiple times, each admission was captured independently. Upon first entry into the study, demographics and past medical history were captured for each child. Thereafter, data pertaining to each admission was captured separately, including initial seizure presentation, referral, treatment, investigations, and length of admission. In addition, the etiology of CSE was recorded and then classified according to the framework proposed by Trinka et al. (2). This included: “Acute,” CSE caused by acute systemic illness or CNS injury (e.g., metabolic or electrolyte abnormalities, infection, stroke); “Remote,” CSE followed previous CNS injury (e.g., post-stroke, post-infective, post-traumatic); “Progressive,” CSE was the result of extending CNS disease (e.g., brain tumor); “Electroclinical,” CSE presenting as part of a defined electroclinical syndrome (e.g., Dravet syndrome); and “Unknown,” cause for CSE not found during admission. However, as it was not possible to perform EEG on all patients, the EEG axis was excluded. Febrile CSE was defined as CSE provoked by hyperthermia (>38.4 degrees Celsius) in the absence of prior afebrile seizures and evidence of acute central nervous system disease (28). Adverse events were defined as an acute decompensation in the child's state that followed the use of antiseizure medication, typically in the form of respiratory depression and or hypotension. All children presented in status are managed in an acute care setting where they are monitored for adverse events as part of routine emergency care. If these became evident, appropriate resuscitation and respiratory support measures were taken (e.g., inotropic support, assisted ventilation) and, when necessary, the child was referred to the PICU for further monitoring and management.

### Admission Procedure

Children with CSE presenting to the RCWMCH were admitted to the medical emergency unit where they received standard monitoring and airway protection. This included continuous monitoring of heart rate, respiration, blood pressure and peripheral temperature as well as point-of-care tests such as urinalysis, glucose and blood gas analysis (including oxygen saturation). In addition, routine blood samples were sent to the laboratory including inflammatory markers and electrolytes. Additional blood investigations were performed as required. Children received urgent computed tomography (CT) imaging for focal seizures without a known cause or in the presence of focal neurological deficit, signs of raised intracranial pressure and/or prolonged depressed level of consciousness following seizure termination (20). Magnetic resonance imaging (MRI) was not performed in the acute setting. Where indicated, lumbar puncture was performed once the patient was stabilized and raised intracranial pressure excluded.

### Treatment Protocols

**Figure 1:**
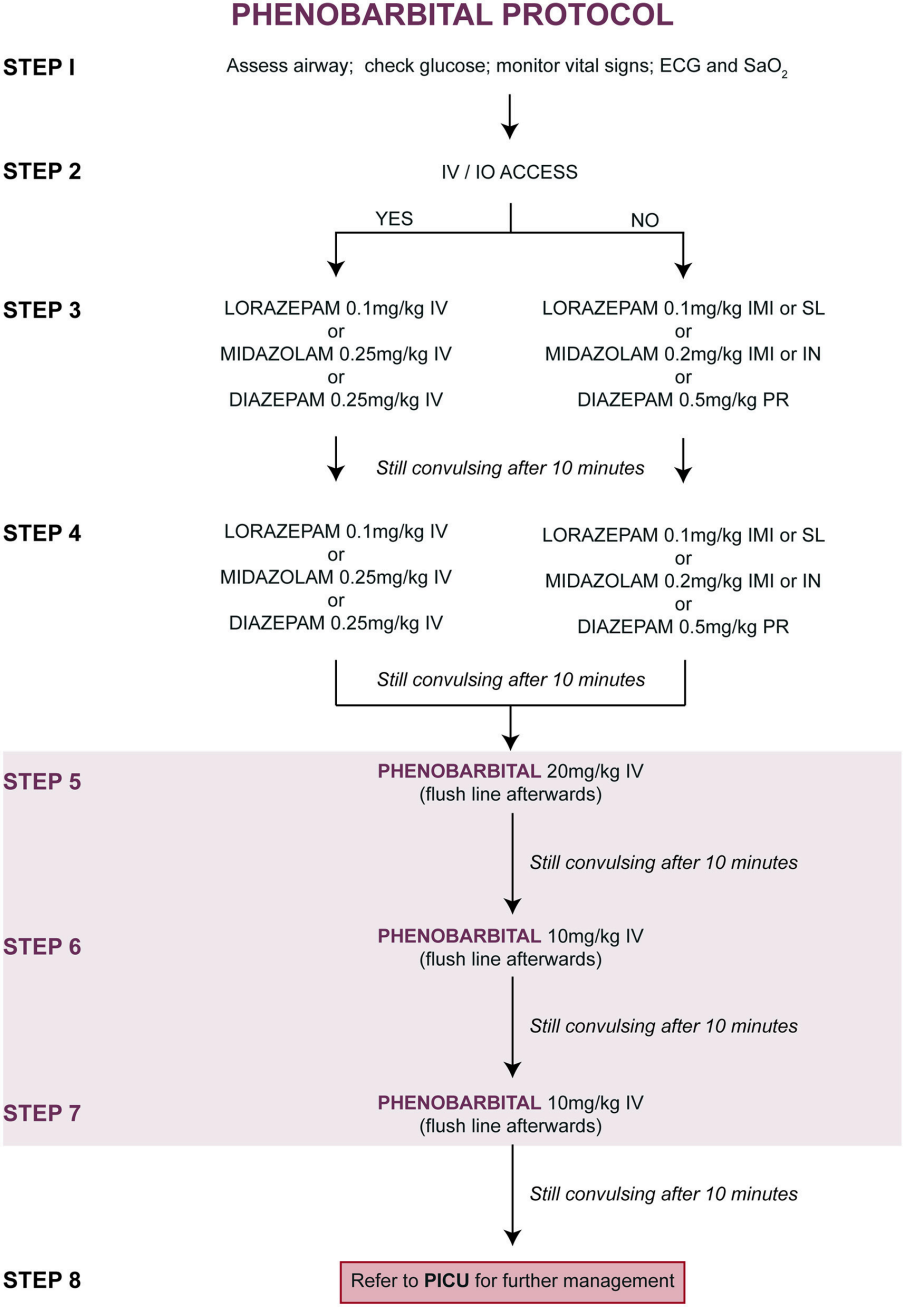
Treatment of convulsive status epilepticus (CSE): parenteral phenobarbital protocol. ECG, electrocardiogram; IMI, intramuscular injection; IN, intranasal; IV, intravenous injection; PR, per rectum; PICU, pediatric intensive care unit; SaO2, oxygen saturation; SL, sublingual.

**Figure 2:**
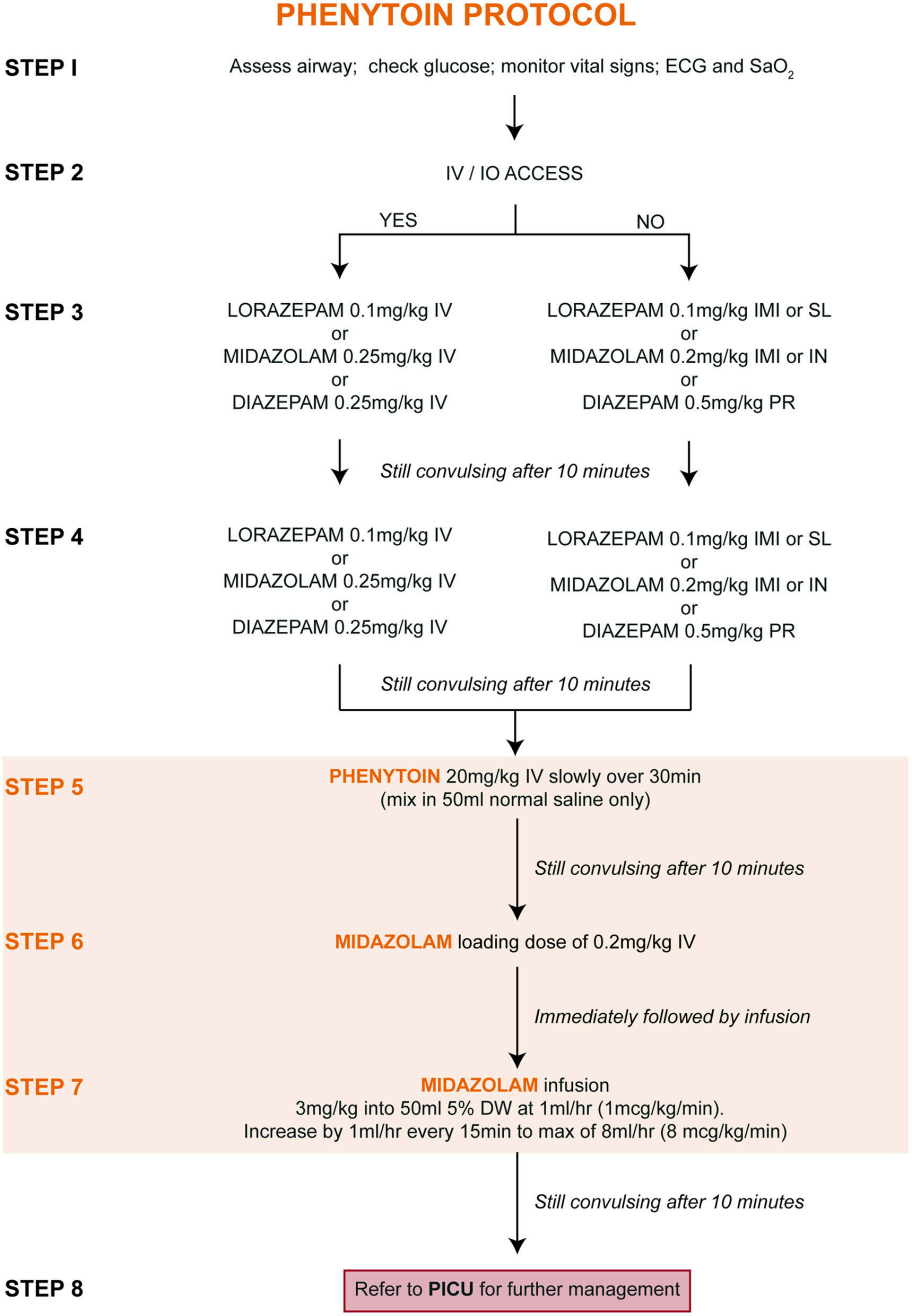
Treatment of convulsive status epilepticus (CSE): parenteral phenytoin protocol. DW, dextrose water; ECG, electrocardiogram; IMI, intramuscular injection; IN, intranasal; IV, intravenous injection; PR, per rectum; PICU, pediatric intensive care unit; SaO2, oxygen saturation; SL, sublingual.

Upon entry into the study, children were randomly allocated (at a ratio of 1:1) to one of two treatment protocols [Figure 1 (PHB) and Figure 2 (PHY)]. These protocols are based on the Emergency Triage Assessment and Treatment (ETAT) guidelines and used in the sub-Saharan African setting for the management of SE (20). Pre-hospital intravenous administration of benzodiazepines by emergency services were included, however all other routes of administration were not counted due to the lack of consistency in their administration. Children who did not respond to either the PHB or the PHY treatment protocols were referred to the PICU. Other reasons for admission to the PICU included respiratory depression following administration of the second-line agent, need for inotropic support, etiology-related concerns requiring intensive monitoring (e.g., severe electrolyte imbalances) and or prolonged state of a depressed level of consciousness.

### Outcome Measures

We focused on short-term outcomes when comparing the response to the administration of the different second-line treatments given to patients with refractory CSE (i.e., who did not respond to first-line benzodiazepines). We performed an intention to treat (ITT) analysis that includes all patients that were randomized and allocated a treatment (29). All randomized patients were included in the analysis regardless of whether they received the allocated treatment. This approach was used to minimize the effect of protocol deviations as well as patients not progressing to second-line therapy on the random assignment of protocols. We do acknowledge that this approach only allows for a conservative measure of the effect of the treatments, but more accurately accounts for the inconsistencies that are inherent in clinical practice. The primary outcome was the success of the second-line agent in terminating refractory CSE. Specifically, we compared how many episodes of refractory CSE were terminated after a single dose of parenteral PHB or PHY. Secondary outcomes included the need for PICU admission and seizure recurrence (termed “breakthrough seizures”) within the first 24 h following termination of CSE. Furthermore, we calculated the number needed to treat (NNT) to show how many patients with refractory CSE would need to have followed the parenteral PHB protocols over the parenteral PHY protocol to prevent admission to the PICU (calculation performed using ClinCalc (30)).

### Sample Size

There is limited evidence quantifying the effectiveness of parenteral PHB and PHY for terminating pediatric CSE. The evidence available suggests second-line treatment with a single dose of parenteral PHB is effective in terminating pediatric CSE in 77% of cases (24). In contrast, Rai et al. (31) show that second-line treatment with PHY is effective in 97% of cases. We therefore based our estimated incidence of the primary outcome, termination of CSE, as 77% for the PHB group and 97% for the PHY group. We used ClinCalc (30) to calculate sample size with an independent dichotomous endpoint (two-sided test) using a Type I error probability (α) set at 0.05 and a Type II error probability (β) set at 0.2 (power 80%). The calculated sample size to see a difference in efficacy between the second-line agents was 86, with 43 episodes required in each of the two treatment groups. However, as the supply of parenteral PHB became limited, the study was stopped before the desired sample size was achieved. A *post-hoc* power calculation was performed to measure the statistical power with the acquired sample size. As response to a particular second-line agent was our primary outcome, after study completion we performed a *post-hoc* power analysis to recalculate the actual statistical power against what we had expected (using the ClinCalc (30)). As the actual incidence of CSE termination was 86% for patients who received PHB compared to 45% for those who received PHY, the *post-hoc* power was 94% which is higher than the 80% we had originally calculated whilst designing this study.

### Randomization, Blinding and Concealment

Randomization of protocols was performed using a Research Randomizer (32) using a simple randomization technique (33). Study protocols were prepared in sealed, opaque envelopes by RJB and the numbers allocated by a separate party who was not otherwise involved in the study. Envelopes were placed in a secure box in the medical emergency unit. On admission into the study, the attending doctor managing an eligible patient took an envelope containing the protocol that would then be assigned to that patient. Due to differences in administration, the doctors implementing the protocols could not be blinded. However, after completion of patient recruitment and data collection, all patient identifiers were removed. In addition, those patients who had received second-line therapy were kept unknown until after the data analysis was performed.

### Follow-Up

Patients were followed-up throughout their admission. Formal measurements of long-term outcomes (including neurodevelopmental assessment) are intended for a future follow-up study.

### Data Analysis

Data were analyzed using custom scripts written on MATLAB (Statistics Toolbox, Release 2018a The MathWorks, Inc., Natick, Massachusetts, United States). We used a pair-wise deletion to deal with missing data that was not present in the patient's records. For continuous data, normality was established using the Shapiro-Wilk test and thereafter parametric (i.e., *paired* or *unpaired student's t-tests*) or nonparametric tests (i.e., *Mann-Whitney U-test*) were performed. Data that were not normally distributed were reported as median with the interquartile range (*IQR*). Categorical data were summarized in contingency tables with differences between groups identified using the *Fisher-exact* or *chi-squared* (*X*^2^) tests. Contingency tables were used to calculate associations between exposures (i.e., treatments received) and outcomes (i.e., success in terminating CSE, need for admission to PICU, etc.). Associations are reported as relative risk (RR) with its associated 95% confidence interval. Significance was defined as *p* < 0.05.

## Results

**Figure 3:**
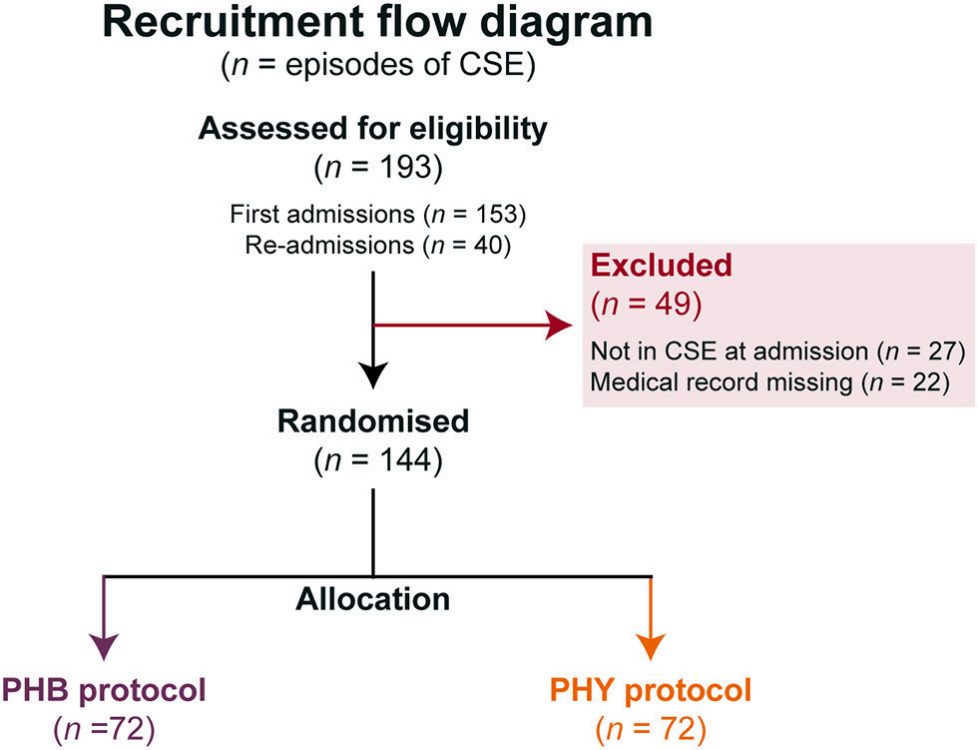
Flow chart of protocol allocation. Number of episodes of pediatric convulsive status epilepticus (*n*) studied and allocation to the different second-line treatment protocols. All patients allocated a pathway were included in the intention-to-treat analysis. BZP, benzodiazepines (including diazepam, lorazepam and midazolam); CSE, convulsive status epilepticus; PHB, phenobarbital; PHY, phenytoin.

Over a three-year period, a total of 193 episodes of CSE were entered into the study with 40 of these being re-admissions (Figure 3). Forty-nine episodes needed to be excluded as they either did not meet the definition of CSE at admission or their medical records could not be found to complete data collection. This left a total of 144 episodes from 111 patients. There was a 50:50 split between the children that were allocated to either the PHB or PHY protocol (72 episodes in each group). All episodes of the CSE in these two groups were included in the intention-to-treat analysis. The demographic and past medical history for both the total patient cohort (*n* = 111) and each treatment arm is shown in Table 1. Of the full cohort, 46% of children had previously been admitted for seizures and 42% had a preceding diagnosis of epilepsy. In those children with epilepsy, structural (53%) and genetic (26%) causes were the most common.

**Table 1:** Demographics of patient cohort recruited into study.

	**Full cohort (*n* = 111)**	**PHB protocol (*n* = 52)**	**PHY protocol (*n* = 59)**	***p***
**GENDER**				
Female	58 (52.3%)	26 (50.0%)	32 (54.2%)	0.71
Male	53 (47.7%)	26 (50.0%)	27 (45.8%)	
**PAST MEDICAL HISTORY**				
HIV-infected	3 (2.7%)	2 (3.8%)	1 (1.7%)	0.60
Cerebral palsy	20 (18.0%)	10 (19.2%)	10 (16.9%)	0.75
Previous TBM	3 (2.7%)	1 (1.9%)	1 (1.7%)	>0.99
Previous TBI	2 (1.8%)	1 (1.9%)	2 (3.4)	>0.99
**NEONATAL HISTORY**				
Pre-term (< 37 weeks)	18 (16.2%)	7 (13.5%)	8 (13.5%)	0.76
Documented HIE	10 (9.0%)	4 (7.7%)	6 (10.2%)	0.74
**SEIZURE HISTORY**				
Previous admission for seizures	51 (45.9%)	28 (53.8%)	23 (39.0%)	0.13
Confirmed epilepsy diagnosis	47 (42.3%)	26 (50.0%)	21 (35.6%)	0.18
**EPILEPSY ETIOLOGY**				
Genetic	12 (25.5%)	7 (26.9%)	5 (23.8%)	0.54
Infectious	1 (2.1%)	1 (3.8%)	–	0.47
Structural	25 (53.2%)	12 (46.2%)	13 (61.9%)	>0.99
Unknown	9 (19.2%)	6 (23.1%)	3 (14.3%)	0.3

*HIE, hypoxic ischemic encephalopathy; HIV, human immune-deficiency virus; TBI, traumatic brain injury; TBM, tuberculous meningitis*.

In terms of overall presentation of pediatric CSE (Table 2), the most common seizure semiology was generalized CSE (65%) due to an acute etiology (60%). At the time of admission, the median age for the full cohort was 28.1 months (*IQR* 15.5–66.01). There was a near equal prevalence of continuous (51%) vs. intermittent episodes (49%) of CSE. In addition, 19% of admissions met the diagnostic criteria for FSE. Between the patients randomized to the PHB and PHY treatment groups, there were no differences in patient demographics nor in the presentation of CSE.

**Table 2:** Overview of presentation of CSE episodes included in this study.

	**Total episodes (*n* = 144)**	**PHB protocol (*n* = 72)**	**PHY protocol (*n* = 72)**	***p***
**AXIS I: SEMIOLOGY**				
Focal onset evolving into bilateral SE	28 (19.4%)	13 (18.1%)	15 (20.8%)	0.83
Generalized	93 (64.6%)	46 (63.9%)	47 (65.3%)	>0.99
Unknown focal or generalized	23 (16.0%)	13 (18.1%)	10 (13.9%)	0.65
**TYPE Of CSE**				
Continuous	73 (50.7%)	40 (55.6%)	38 (52.8%)	0.87
Intermittent	71 (49.3%)	32 (44.4%)	34 (47.2%)	
**AXIS II: ETIOLOGY**				
Acute	86 (59.7%)	45 (62.5%	41 (56.9%)	0.93
Electroclinical	20 (13.9%)	10 (13.9%)	10 (13.9%)	>0.99
Remote	24 (16.7%)	13 (18.1%)	11 (15.3%)	0.82
Unknown	14 (9.7%)	4 (5.6%)	10 (13.9%)	0.16
**Febrile status epilepticus**	27 (18.8%)	14 (19.4%)	13 (18.1%)	>0.99
**AXIS IV: AGE**				
Age at admission- median months (*IQR*)	28.1 (15.5–66.0)	25.7 (13.1–65.6)	22.2 (14.8–46.3)	0.48
Infancy (1 month−1 year)	25 (17.4%)	9 (12.5%)	16 (22.2%)	0.19
Childhood (>1 year−12 years)	119 (82.6%)	63 (87.5%)	56 (77.8%)	

*SE, status epilepticus; CSE, convulsive status epilepticus*.

**Figure 4:**
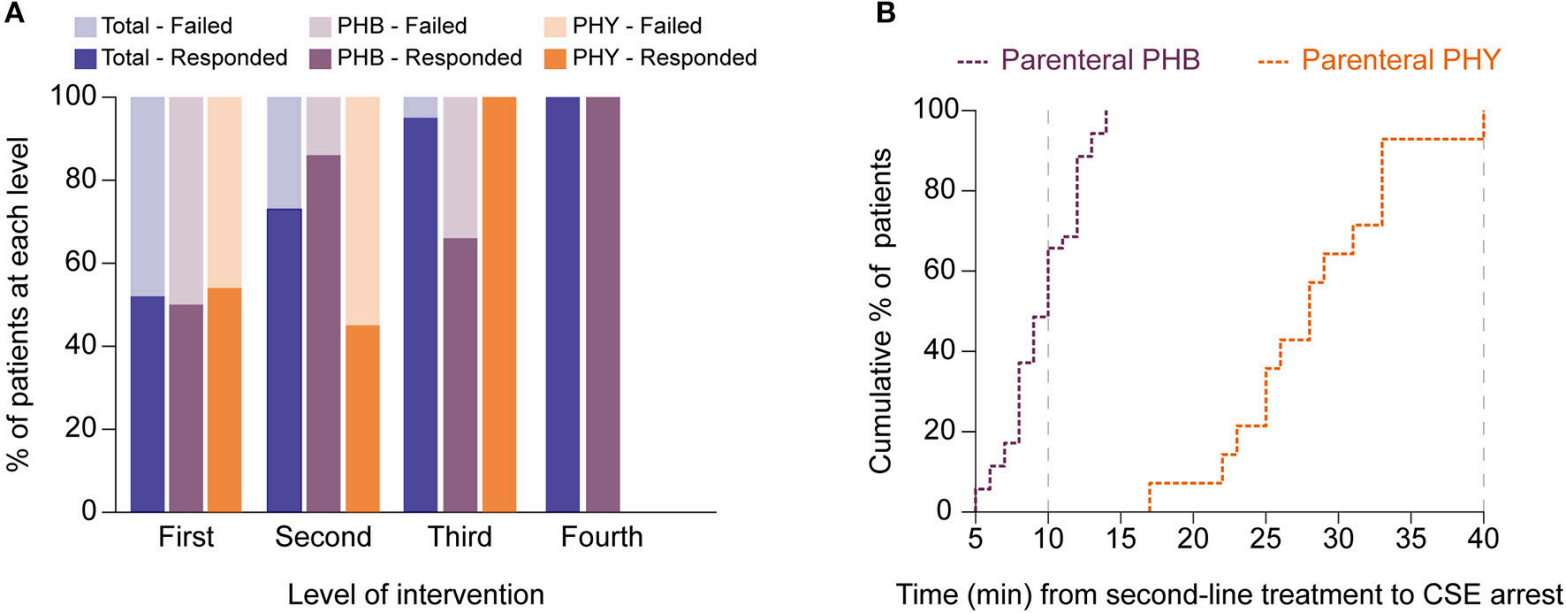
Second-line treatment with parenteral phenobarbital terminates pediatric convulsive status epilepticus more successfully and faster than parenteral phenytoin. **(A)** Bar graph showing differences in responses to different levels of intervention between the total cohort (blue) as well as for the phenobarbital (PHB, purple) and phenytoin (PHY, orange) treatment groups. Dark shading indicates percentage of patients that responded to level whilst lighter shading shows those patients who failed that level. **(B)** Cumulative percentage plot showing the proportion of patients who responded (i.e., CSE terminated) to either parenteral phenobarbital (purple) vs. parenteral phenytoin (orange) at specific time points (in minutes) after the second-line agent was introduced. Gray dashed lines indicate when the protocol recommends progression to a third-line agent. Y axis represents the cumulative percentage of patients whose CSE had terminated whilst X axis represents time in minutes.

Looking at management, overall 48% required second-line intervention with a further 13% requiring third-line intervention (Table 3 and Figure 4A). The median time to first-line treatment was 50 min (*IQR* 33.8–70.5). Overall, 20% of children presenting in CSE required admission to the PICU mostly due to concerns of respiratory depression (66%). Breakthrough seizures occurred in 13% of patients.

**Table 3:** Overall management of CSE and differences in outcomes between PHB and PHY groups.

	**Total episodes (*n* = 144)**	**PHB protocol (*n* = 72)**	**PHY protocol (*n* = 72)**	***p***
**RESPONSE TO TREATMENT (RESPONDED/TOTAL)**				
First-line: benzodiazepines	75/144 (52.1%)	36/72 (50.0%)	39/72 (54.2%)	0.74
Second-line: PHB or PHY	50/69 (72.5%)	31/36 (86.1%)	15/33 (45.5%)	0.0003
Third-line: repeated PHB or MDZ	18/19 (94.7%)	4/5(66.7%)	18/18 (100%)	0.002
Fourth-line: PICU	1 (100%)	1/1 (100%)	-	>0.99
**TREATMENT TIMES (MINUTES)**				
Onset to admission	40.0 (25.0–65.0)	35.0 (25.0–60.0)	40.0 (22.8–65.0)	0.99
Admission to treatment	5.0 (5.0–10.0)	5.0 (5.0–10.0)	5.0 (5.0–10.0)	0.61
Onset to first-line treatment	50.0 (33.8–70.5)	50.0 (34.0–72.0)	50.0 (32.0–70.0)	0.83
Total CSE duration	73.0 (48.0–109.0)	64.0 (45.0–103.5)	83.0 (53.0–115.0)	0.04
First-line treatment to arrest	16.5 (5.0–45.0)	5.0 (3.0–12.0)	9.0 (3.0–14.0)	0.29
Second-line treatment to arrest	28.0 (13.3–41.5)	10 (10.0–21.8)	28.0 (24.5–33.0)	< 0.0001
Third-line treatment to arrest	13.0 (8.0–25.0)	13.0 (13.0–28.0)	12.0 (6.5–22.0)	0.39
**PICU**				
Required admission	29 (20.1%)	9 (12.5%)	20 (27.8%)	0.04
**REASONS FOR PICU ADMISSION**				
Inotropic support needed	2 (6.9%)	–	2 (10.0%)	>0.99
Respiratory depression	19 (65.5%)	5 (55.6%)	14 (70.0%)	0.68
Prolonged LOC	7 (24.1%)	3 (33.3%)	4 (20.0%)	0.64
Seizure control	1 (3.5%)	1 (11.1%)	–	0.31
**Breakthrough seizures**	18 (12.5%)	8 (11.1%)	10 (13.9%)	0.8

*LOC, prolonged loss of consciousness; PICU, pediatric intensive care unit*.

With regards to comparing the two second-line treatment protocols (Table 3), of the 72 episodes allocated in each group, only 36 episodes (50%) progressed to second-line intervention in the PHB group and 33 (46%) in the PHY group. Of the patients who required second-line treatment, we found after a single dose of parenteral PHB, 86% of children responded with the CSE termination. In contrast, CSE was terminated in only 46% of children who received a single parenteral dose of PHY. Furthermore, we noted that in the patients who required third-line treatment, 67% of patients who responded to repeated parenteral boluses of PHB with one patient requiring fourth-line intervention given in the PICU. In contrast, 100% of patients given the MDZ infusion responded.

To measure the efficacy of a particular treatment, we used contingency tables to compare the number of children who had their refractory CSE successfully terminated using second-line treatment with either PHB or PHY. Using this approach we were then able to calculate the relative risk (*RR*) of failing second-line treatment (i.e., CSE not terminating within 10 min after the full dose of the agent had been given). We found that patients who received parenteral PHB as second-line treatment had a significantly lower risk of needing third-line intervention compared to those who received PHY (14 vs. 54%, *RR* = 0.3, *95% CI* 0.1–0.7, *p* = 0.0003). Furthermore, in the patients who responded to second-line treatment, we found that PHB terminated CSE significantly faster compared to PHY (*median* 10 min, *IQR* 10.0–21.8 vs. *median* 28.0 min, *IQR* 24.5–33.0, *p* < 0.0001; Figure 4B). Of those patients who did respond to third-line treatment, there was no difference in time to CSE arrest between those that received repeated PHB boluses or the MDZ infusion (*median* 13.0 min, *IQR* 13.0–28.0 vs. *median* 12.0, *IQR* 6.5–22.0, *p* = 0.4).

We also noticed that overall fewer patients allocated to the PHB protocol required admission to the PICU compared to those allocated to the PHY protocol (13 vs. 28%, *RR* = 0.57 *95% CI* 0.32–0.99). We calculated a NNT of 6.5, indicating that 6.5 patients with refractory CSE would have to have followed the parental PHB protocol in order to reduce a single admission to the PICU that would have otherwise occurred if they had followed the parenteral PHY protocol. We then assessed the risk of needing admission to PICU at each level of intervention. Notably we found that in patients who required second-line intervention, there was no significant difference between the PHB and PHY groups (30 vs. 7%, *RR* = 1.45, *95% CI* 1.1–2.0, *p* = 1.4). However, in patients who required third-line treatment, those that received the repeated boluses of parenteral PHB were at a significantly lower risk of requiring PICU admission compared to those that received the MDZ infusion (25 vs. 100%, *RR* = 0.05, *95% CI* 0.01–0.4, *p* = 0.003). Lastly, we saw no difference in the proportion of patients who had breakthrough seizures within the first 24 h after CSE was terminated between the two groups (11 vs. 14%, *p* = 0.8).

## Discussion

In conducting this study, we have been able to describe the local burden of CSE while also providing new data on the efficacy of parenteral PHB as second-line management. A large proportion of our cohort with CSE had previously presented with seizures or had an established diagnosis of epilepsy prior to their entry into the study (Table 1). In contrast, Sadarangani et al. (5) reported only 23% of their Kenyan children presenting in confirmed CSE had a prior history of seizures. The majority of our children with epilepsy had an underlying structural cause. Surprisingly, the proportion of children with a documented past medical history of human immunodeficiency virus (HIV), tuberculous meningitis (TBM), traumatic brain injury (TBI) or hypoxic ischemic encephalopathy (HIE) was relatively small despite the high prevalence of these conditions in the South African context (34, 35). In the emergency setting the past medical history may not have been adequately recorded, leading to possible underestimation of these conditions.

The majority of our cohort presented with generalized CSE (Table 2). While this proportion is similar to that reported by Chin et al. (4) in their UK-based study, we suspect that generalized FSE was overestimated in our cohort due to the high prevalence of structural epilepsies. There may in fact be a much higher burden of seizures with unrecognized focal onset evolving into bilateral SE. The most common cause for pediatric CSE was an acute CNS injury or infection with 19% of the cases being FSE. These figures are again similar to what has been previously reported in both Kenya and the United Kingdom (4, 5).

The management of pediatric CSE in our cohort contrasts similar studies conducted in resource-equipped settings (Table 3). Firstly, the time from onset to admission was higher in our cohort (median 40 min) although we expected there to be a greater delay in our setting given the known barriers to accessing care. Secondly, the time from seizure onset to administration of first-line BZPs was significantly longer in our cohort (median of 50 min compared to 28 min reported in the Chin et al. (13) cohort). Our study was set in an urban environment and we would therefore expect the time from onset to admission to be even longer for children in rural settings, as suggested by previous work done in Kenya (5). Thirdly, the total seizure duration reported in our study was longer (73.5 min compared to 65 min reported in the Chin et al. cohort). Chin et al. (13) have previously identified a lack of prehospital treatment, delayed admission time, more than two benzodiazepines and intermittent CSE as risk factors for CSE lasting longer than 60 min. In addition, we reported a higher proportion of patients who did not respond to first-line benzodiazepines (48 vs. 35% reported in the Chin et al. cohort). The proportion of patients requiring PICU admission (20%) was similar to the Chin et al. cohort (20%). This was surprising, as we expected that the longer delay in treatment would result in a greater need for PICU intervention. However, this may be explained by differences in accessibility to PICU, as in our setting the access to PICU is significantly more limited compared to a more resource-equipped hospital.

Our intention-to-treat comparative analysis has shown that parenteral PHB is a more effective and efficient second-line agent for refractory CSE compared to parenteral PHY. This is evident in PHB terminating CSE faster and more successfully by decreasing the need for higher intervention and admission to PICU. However, we do acknowledge that this difference may have a modest effect clinically as the calculated NNT was 6.5 indicating that for every 6.5 patients treated with the PHB protocol, one patient is prevented from being admitted to the PICU if they otherwise followed the PHY protocol. A major contributing factor to why PHY is less effective than PHB is likely due to the longer time needed to administer it (36). The reason why patients in the PHY group required admission to the PICU was due to increased need for third-line with a midazolam infusion, which is known to cause significant respiratory depression. The need for admission to the PICU is of particular importance due to the lack of PICUs, and PICU beds, available in resource-limited healthcare settings. Furthermore, for the children who required third-line intervention, we found no significant difference in efficacy between using repeated parenteral boluses of PHB vs. using an MDZ infusion.

Previous work by Malamiri et al. (24) from Iran compared parenteral sodium valproate against intravenous phenobarbital as second-line management of pediatric refractory CSE. Their results show that parenteral sodium valproate appears more effective in decreasing recurrence of seizures within 24 h as well as decreasing adverse effects (namely respiratory depression) compared to parenteral phenobarbital. However, parenteral sodium valproate is not commonly used within Africa for the management of pediatric CSE with the common alternative to parenteral phenobarbital being parenteral phenytoin. Apart from our study, there is currently no evidence comparing parenteral phenobarbital vs. phenytoin for second-line management of pediatric CSE. The only direct comparison can be found in the study by Treiman et al. (22) who showed that first-line management with parenteral phenobarbital appeared more effective in terminating CSE in adults. Therefore, for those practicing within sub-Saharan Africa there remains a demand to compare the commonly used parenteral phenobarbital against parenteral phenytoin which remains as the recommended second-line agent on pediatric CSE management guidelines.

Our findings conflict with those of Sreenath et al. (37) who claim that parenteral lorazepam and the combination of diazepam and phenytoin is 100% effective in terminating CSE. Moreover, Rai et al. (31) also suggested that phenytoin is 97% effective in terminating pediatric status epilepticus. To explain this difference in PHY efficacy, it is important to consider differences in the underlying cause of CSE. Specifically, FSE is thought to be associated with sodium-channel mutations that would impact the efficacy of PHY. Notably, PHY was only effective in terminating FSE in 14% of pediatric cases (38). Comparing our study with Sreenath et al. (37), we noted that 27% of the children in our study who received PHY had FSE compared to 7.9% in theirs.

In terms of the generalizability of this study, we believe it provides useful clinical data for other resource-limited healthcare settings. Our data shows that parenteral phenobarbital is an effective antiseizure medication in the management of pediatric CSE, thereby validating previous reports (26, 27). However, the findings of this study may not be globally relevant as in resource-rich settings there is ready access to newer antiseizure medications (namely intravenous levetiracetam). This study is also not comparable to the larger double-blinded randomized controlled studies currently underway (EcLiPSE, ESETT and ConSEPT). Nevertheless, we have been able to demonstrate that within a resource-limited setting, parenteral PHB remains the most effective treatment. While in our setting at RCWMCH we are able to give PHY followed by a midazolam infusion with appropriate monitoring and access to PICU, in the majority of centers across Africa this is not viable. In contrast, PHB is an effective agent for the management of CSE while decreasing demands on healthcare resources. PHB is often quoted to cause hypotension and respiratory depression, but there is little data to support this. Previous work by Crawford et al. (39) reported that even very high doses of parenteral PHB is safe, with few adverse effects. Respiratory depression and hypotension following treatment for CSE were found to be related to confounding factors including excessive levels of benzodiazepines and/or the underlying etiology.

While we attempted to uphold scientific rigor throughout the design and implementation of this study, it is not without limitations. Most notably, the reliability of the reporting of both time of seizure onset and seizure semiology by the child's caregivers should be viewed with caution. During the recruitment of children into this study, our sample size was affected by a large number of exclusions. This was in part due to incorrect diagnosis of CSE, highlighting a need to train local practitioners in the identification of CSE according to the latest guidelines. In addition, 22 children were excluded due to missing paper-based medical records. This reflects a concerning inefficiency within our healthcare system which negatively impacts research efforts and, more importantly, the care of patients. Furthermore, as EEG was not routinely performed in the acute setting, we were not able to exclude non-convulsive SE after CSE was terminated (40). However, standard operating procedure of the neurology service ensures that any child with persistent reduced level of consciousness post seizure termination, or abnormal movements post seizure termination, undergoes EEG. This study was also vulnerable to bias as the practitioners administering the protocols were not blinded. While the analysis was blinded, it was not possible to blind the implementation due to differences in administration of the agents.

## Conclusion

This study has characterized the burden of pediatric CSE within our local setting, providing important epidemiological insight. We hope these findings will be useful for those managing pediatric CSE within resource-limited settings while also advocating for parenteral PHB to be retained.

## Ethics Statement

This study was approved by the University of Cape Town Human Research Ethics Committee (reference number: 297/2005). Signed consent forms were obtained from the parents or legal guardian of all children recruited into this study.

## Author Contributions

RB was responsible for study design, data collection, data analysis, and writing of the manuscript. SA was involved in the study design, data collection and writing of the manuscript. AS-C and AN assisted in study design and data collection. HB was involved in study design, data collection, data analysis, and supervising the writing of the manuscript. JW was the principle investigator and supervised the study design, data collection, data analysis, and writing of the manuscript.

### Conflict of Interest Statement

The authors declare that the research was conducted in the absence of any commercial or financial relationships that could be construed as a potential conflict of interest.
